# Carrying a passenger and relaxation before driving: Classification of young drivers’ physiological activation

**DOI:** 10.14814/phy2.15229

**Published:** 2022-05-18

**Authors:** Quentin Meteier, Marine Capallera, Emmanuel De Salis, Marino Widmer, Leonardo Angelini, Omar Abou Khaled, Elena Mugellini, Andreas Sonderegger

**Affiliations:** ^1^ HumanTech Institute University of Applied Sciences and Arts of Western Switzerland Fribourg Switzerland; ^2^ Haute‐Ecole Arc Ingénierie University of Applied Sciences and Arts of Western Switzerland Saint‐Imier Switzerland; ^3^ Department of Informatics University of Fribourg Fribourg Switzerland; ^4^ Business School Institute for New Work Bern University of Applied Sciences Bern Switzerland

**Keywords:** driver state, machine learning, passenger, physiology, relaxation, stress

## Abstract

Drivers are often held responsible for road crashes. Previous research has shown that stressors such as carrying passengers in the vehicle can be a source of accidents for young drivers. To mitigate this problem, this study investigated whether the presence of a passenger behind the wheel can be predicted using machine learning, based on physiological signals. It also addresses the question whether relaxation before driving can positively influence the driver's state and help controlling the potential negative consequences of stressors. Sixty young participants completed a 10‐min driving simulator session, either alone or with a passenger. Before their driving session, participants spent 10 min relaxing or listening to an audiobook. Physiological signals were recorded throughout the experiment. Results show that drivers experience a higher increase in skin conductance when driving with a passenger, which can be predicted with 90%‐accuracy by a k‐nearest neighbors classifier. This might be a possible explanation for increased risk taking in this age group. Besides, the practice of relaxation can be predicted with 80% accuracy using a neural network. According to the statistical analysis, the potential beneficial effect of relaxation did not carry out on the driver's physiological state while driving, although machine learning techniques revealed that participants who exercised relaxation before driving could be recognized with 70% accuracy. Analysis of physiological characteristics after classification revealed several relevant physiological indicators associated with the presence of a passenger and relaxation.

## Graphical abstract

This work contains a classification of the driver's condition (driving with a passenger or not and performing relaxation before driving or not), based on physiological indicators calculated from raw signals. Machine learning classification procedures revealed that the presence of a passenger can be predicted with a 90%‐accuracy, while the practice of relaxation can be predicted with an 80%‐accuracy.

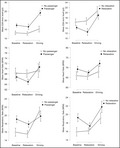

## 1 INTRODUCTION

The Organization for Economic Co‐operation and Development (OECD) noted that young people under the age of 25 years represent about 10% of the population in OECD countries, but account for more than 25% of drivers killed on the road (OECD, 2006), indicating the importance to focus on this population with regard to traffic safety. In this regard, driving‐related stress has been shown to represent an important factor for drivers’ safety and individual well‐being (Matthews et al., 1998). Experience of stress while driving is influenced by various factors, such as the driving situation (e.g., highway or urban area; Healey & Picard, 2005), adverse weather conditions (Tavakoli et al., 2020), or the presence of a passenger (Chung et al., 2019). Previous research has shown that the presence of passengers in the car is related to risky driving behavior and higher accident rates, especially for young drivers (Chen, 2000).

Despite the potential important influence of passenger presence for road safety, there is relatively little empirical evidence addressing this phenomenon. A focus on physiological indicators might be of interest in order to obtain a better understanding of the causes and the cause–effect relationship in this regard. In addition to a better understanding of the potential stressful link between passenger presence and driving, it is important to address the question how to overcome such stressful situations. Various stress management techniques have positive effects on measures of well‐being and health (Varvogli & Darviri, 2011). In particular, meditation and relaxation have shown to be very effective in reducing stress (Davis et al., 2008). However, the effectiveness of such techniques in the driving context has yet to be addressed.

In the wake of an increased automation of the transportation sector, it is conceivable that, based on physiological indicators, the automatic detection of potentially dangerous internal conditions such as arousal may be of interest in the future. In this regard, models and methods should be developed to evaluate them empirically. The goal of this study is to evaluate the influence of the presence of a passenger and relaxation on a driver's physiological state, and to develop and evaluate a method based on machine learning that automatically detects drivers’ physiological activation related to these.

### 1.1 The presence of a passenger as a factor of stress

While the presence of a passenger can have safety‐critically influences in young drivers, this was not the case for older drivers, who showed an inversed effect pattern when driving with passengers (i.e., a reduction in accidents; Chen, 2000). This can be partly explained by the fact that young drivers are more prone to show risk‐taking (Centifanti et al., 2016; Ulleberg, 2004), which may be linked with the driver's affective state (Chliaoutakis et al., 2002; Megías et al., 2011). In a similar vein, the lack of experience of young drivers might lead to an increased level of stress when passengers are present.

Being observed while performing a task represents a source of stress (Schrier, 1992), even if the observers do not interact with the observee (Sonderegger & Sauer, 2009). This stress response affects emotional state and influences performance (Sonderegger & Sauer, 2009), although contrasting results have been reported in this regard (Larkin et al., 1998), which might be explained by social facilitation theory (Zajonc, 1965). According to this theory, the presence of others is linked with an increase in arousal. This increase has a positive influence on the performance of simple and well‐learned tasks, while it impinges on the performance of complex and difficult tasks. Since driving can be considered a complex and difficult task for inexperienced and young drivers, it can be assumed that the situation caused by the presence of a passenger leads to increased arousal and stress and thus to a reduction in driving performance. Previous research has indicated that such a stress response can be assessed by measures of heart rate variability (Sonderegger & Sauer, 2009) or systolic blood pressure (Larkin et al., 1998).

### 1.2 Physiological indicators as measures of stress and arousal

Previous research has shown that physiological signals can be used to assess various levels of driving stress (Chen et al., 2017; Healey & Picard, 2005). With recent advances in the development of wearable sensors (e.g., smartwatches or smart clothing), these signals can be collected with high accuracy in a non‐intrusive manner (Merritt et al., 2009; Poh et al., 2010).

Electrodermal activity (EDA) is a signal widely used in research to measure physiological arousal and stress, since eccrine sweat glands, particularly those on the palms and soles of the feet, are highly sensitive to psychologically driven stimuli (Boucsein, 2012; Poh et al., 2010; Reinhardt et al., 2012; Sano & Picard, 2013; Taylor & Machado‐Moreira, 2013). In traffic research, EDA has been used to assess the activation state of drivers (Cacioppo et al., 2007). Features calculated from the raw EDA signal have been shown to be effective for predicting various levels of stress elicited by the driving environment (Bitkina et al., 2019; Chen et al., 2017; Healey & Picard, 2005; Liu & Du, 2018).

The electrocardiogram (ECG) is another physiological signal used to evaluate the driver's state. Heart rate and heart rate variability (HRV) can be used to assess the stress level of individuals, especially when they are under some form of social stress (Kirschbaum et al., 1993; Loeffler et al., 2017). In the context of driving, these indicators are also correlated with stress induced by the driving environment (Chen et al., 2017; Healey & Picard, 2005; Munla et al., 2015; Wang et al., 2013).

Finally, respiration is also used to assess driver state. Respiratory activity is expected to be regular in calm conditions but can be disrupted in stressful situations (Cacioppo et al., 2007). The latter can alter respiratory behavior, causing an increase in respiratory rate, potentially leading to hyperventilation (Grossman, 1983; Suess et al., 1980). However, indicators of respiratory variability have shown in some studies to be less effective in measuring an individual's stress response compared to other physiological indicators (Chen et al., 2017; Healey & Picard, 2005).

### 1.3 Meditation and relaxation as methods to reduce stress

As meditation and relaxation are considered efficient to reduce stress (Sedlmeier et al., 2012), one might expect the application of such techniques to be beneficial in the context of driving. Meditation can be defined as a sequence of steps to achieve an enhanced mental state, also commonly referred to as a meditative state, through various existing methods that utilize specific cognitive strategies (Nash et al., 2013). While meditation is typically practiced over longer period of time (e.g., from several weeks to several years, Goyal et al., 2014), relaxation can include short‐term exercises that do not require a specific level of expertise and experience, aiming to release bodily tension and leading to a psychophysiological state of reduced arousal (Jain et al., 2007).

Various techniques for relaxation and meditation have been developed, ranging from ancient traditional spiritual practices to more recent techniques including mindfulness‐based methods (Henchoz et al., 2021). Many of these techniques have been shown to reduce depression, anxiety, stress, while improving mood and quality of life of individuals (Goldberg et al., 2019; Goyal et al., 2014; Hofmann et al., 2010; Jain et al., 2007; Khoury et al., 2017; Niazi & Niazi, 2011). Meditation can also positively impact a person's physiological state, characterized by short‐ and long‐term decrease in heart rate (Ditto et al., 2006; Solberg et al., 2004) as well as an increase in HRV indicators (Ditto et al., 2006). Meditation can thus have beneficial effects not only during a stressful period, but also after experiencing stress. Even in novices, it has been shown that practicing meditation after a stressful experience leads to a faster decrease in skin conductance and an increase in positive affect (Borchardt & Zoccola, 2018).

In the context of driving, there are few empirical studies on the utility of relaxation and meditation techniques. Barnes and colleagues (2001) evaluated the usefulness of transcendental meditation on cardiovascular function and used a driving task as an experimental stressor. Their results showed a beneficial effect of meditation on participants’ stress responses (including heart rate) after driving. In addition, mindfulness interventions, including relaxation and cognitive change exercises, have been successfully administered to angry drivers (Deffenbacher, 2016).

### 1.4 The present study

The main objective of this study was to assess the influence of the presence of a passenger on the physiological state of drivers. The second goal of this study was to investigate whether pre‐driving relaxation can be used as a technique to mitigate physiological activation due to the presence of a passenger. This work aims to help develop intelligent systems able to recognize the drivers’ state and thus optimally support them (Nguyen et al., 2021).

To address these questions, the presence of a passenger and relaxation prior to driving were manipulated experimentally in a laboratory‐based driving simulation study. ECG, EDA, and respiration were used for continuous monitoring of drivers’ physiological activation. In the perspective of potential use in real conditions, machine learning techniques were used to investigate if driver's physiological activation can be accurately detected in such situations. In this regard, three different classifiers were trained to predict drivers’ condition using a 5‐repeated 4‐fold cross‐validation approach and a large range of features extracted from physiological signals. To understand the models’ decision and find out the most relevant physiological indicators linked with the presence of a passenger while driving and relaxation, a post hoc calculation of feature importance was conducted.

Based on findings of previous research reported above, we expected that driving with a passenger negatively influences drivers’ state for both subjective measure (affective state) and objective measures (physiological indicators) (Healey & Picard, 2005; Kirschbaum et al., 1993; Poh et al., 2010; Reinhardt et al., 2012). Despite the sparse empirical evidence in the context of driving, a brief pre‐driving relaxation is expected to have a positive effect on the driver's physiological and affective state during and after driving (lower physiological activation and negative affect) (Borchardt & Zoccola, 2018; Ditto et al., 2006; Hill & Boyle, 2007; Solberg et al., 2004).

## 2 MATERIAL AND METHODS

### 2.1 Participants

Sixty young participants (22 ± 1.9 years old) were recruited for this study. The sample consisted of 26 male and 34 female students. A specific sample of young drivers has been recruited because the presence of a passenger is particularly dangerous for this age group and negatively influences driving behavior (Chen, 2000). All participants were required to hold a driving license and be of good general health. Participants received course credit for their participation. The study procedure followed the tenets of the Helsinki agreement and written informed consent was obtained from all participants.

### 2.2 Experimental design

The experiment followed a 2 × 2 × 3 mixed design, with driving condition (passenger vs. alone) and relaxation (meditation podcast vs. audiobook) as between‐subjects variables, and measurement time (baseline vs. relaxation vs. driving) as a within‐subjects variable.

### 2.3 Material and instruments

The experiment was conducted in a fixed‐base simulator, composed of two adjacent car seats, a steering wheel (Logitech G27), and pedals (gas and brake) installed in front of a large screen. The driving simulation was back‐projected using a projector (Epsilon EH‐TW3200). Participants were able to adjust the position and inclination of the seat. Two speakers located behind the seats played the sound of the driving simulation to immerse drivers in the driving environment. The free version of OpenDS was used as simulation software.

Physiological signals of drivers were recorded using the Biopac MP36 hardware at a sample rate of 1000 Hz. A digital low‐pass filter with a frequency of 66.5 Hz, a Q factor of 0.5, and a gain of 2000 was applied to all signals. An additional gain of 2000 and 1000 was, respectively, applied to digital filters for the EDA and respiration signals. Lead sets (SS57LA and SS2LB, Biopac) with disposable Ag/AgCl pre‐gelled electrodes (EL507 and EL503, Biopac) were, respectively, used to record the EDA and ECG of participants. Electrodes recording the EDA signal were placed on the distal phalanges of the middle and ring fingers of the non‐dominant hand of participants. The SS5LB respiratory effort transducer (Biopac) recorded the respiration via chest expansion and contraction.

A guided‐mindfulness meditation podcast was used for the manipulation of relaxation and an online audiobook (Sherlock Holmes ‐ Die drei Studenten (Hörbuch), YouTube) was used for the control condition, similar to Ditto et al. (2006) or Borchardt and Zoccola (2018). Audio files were presented via headphones (SONY WH‐1000X M3).

### 2.4 Measures

**FIGURE 1:**
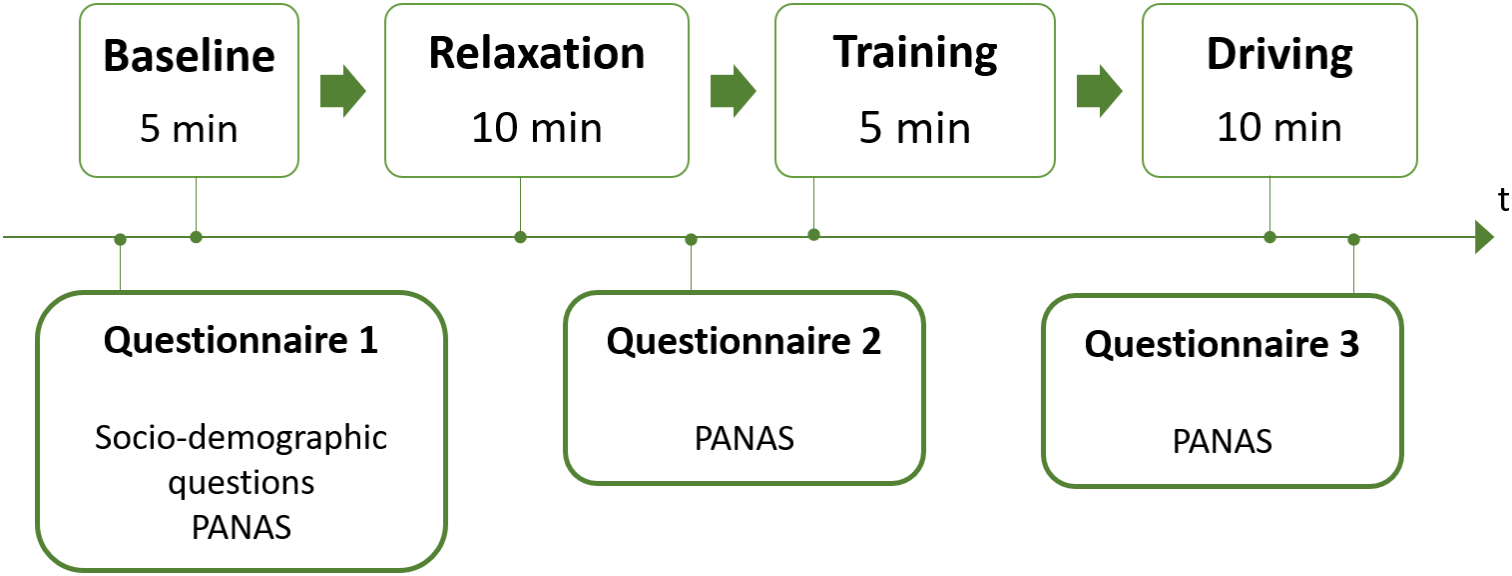
Procedure of the study

The affective state was assessed three times (see Figure 1) via the Positive and Negative Affect Schedule (PANAS; Watson et al. (1988)), which was presented on a tablet. This self‐report questionnaire consists of 20 items with each item rated on a five‐point Likert scale to measure both positive and negative affect as two dimensions of the construct (10 items each).

ECG, EDA, and respiration were recorded throughout the entire experiment. From these signals, physiological indicators corresponding to the different periods of the experiment were calculated: baseline, relaxing or listening to an audiobook, and driving.

### 2.5 Experimental procedure

An overview of the experimental procedure is shown in Figure 1. After being welcomed, participants answered a questionnaire containing socio‐demographic questions (i.e., age, gender, driving experience, driving habits, etc..) and assessed their current affective state (baseline affect). Electrodes were attached and participants were asked to take a seat in the driving simulator. The experiment was composed of four phases: the baseline, the relaxation, the training, and the driving phase.

In the first phase, participants listened to an audiobook for 5 min in order to record the individual baseline of physiological signals. In the second phase, participants either listened to a guided mindfulness‐meditation podcast (relaxation group) or went on listening to the audiobook (control group) for 10 min. Given the short duration of the manipulation with regard to meditation, this phase is referred to as a relaxation phase in the rest of the manuscript. At the end of this phase, the affective state was assessed a second time. The third phase was the driving practice session. It lasted 5 min and gave participants the possibility to familiarize themselves with the driving simulator. Data from the training drive were not considered for analysis.

The fourth phase was the main driving session. To avoid learning effects, different scenarios were used in the driving and training phases. The scenario consisted of a 2 × 2 lane highway without traffic, with repeatedly occurring construction zones on the right lane. Participants had to drive for 10 min. Before driving, participants were instructed to cover the longest distance possible by respecting all traffic regulations and speed limits. Depending on the experimental condition, participants were driving either alone or with a passenger. Trained actors (male and female, randomly assigned to the various experimental conditions) played the passenger, following a script of non‐intrusive conversation with the driver. Participants did not know that the passenger was an actor but thought that the person was also recruited for the experiment. A fictitious draw was used at the beginning of the experiment to assign the roles of the driver and the passenger—in which always the participant was assigned the driver's role. Participants were allowed to talk to the passenger. At the end of the driving task, the affective state was assessed again. Finally, the electrodes were removed and the participant was thanked and discharged.

### 2.6 Classification of drivers’ condition

Three classification tasks were implemented and evaluated in this study:

- **Classification task 1:** The presence of a passenger while driving (passenger vs. no passenger) based on physiological features calculated during the driving session.
- **Classification task 2:** Relaxation practice (relaxation vs. audiobook) based on physiological features calculated during the relaxation phase.
- **Classification task 3:** Relaxation practice before driving (relaxation vs. audiobook) based on physiological features calculated during the driving session.

For every classification task, the dataset contained 55 samples with balanced classes (passenger vs. alone and relaxation vs. audiobook).

The physiological signals collected during the experiment were processed using the Neurokit library in Python (Makowski et al., 2020). Two hundred and twenty four features from 112 indicators (10 from EDA, 74 from ECG, 21 from RESP, 7 from RSA) were calculated from the three raw signals, as presented in Table 1. Indicators of respiratory sinus arrhythmia (RSA) were calculated from processed ECG and respiration signals (Lewis et al., 2012). To consider drivers’ physiological state at rest (baseline) in the training process, two features were calculated for each physiological indicator:

- The indicator's value while relaxing (Task 2) or driving (Tasks 1 and 3)
- The difference of the indicator's value between the driving phase and the baseline phase (∆ Driving‐Baseline) for Tasks 1 and 3 or between the relaxation and the baseline phase (∆ Relaxation‐Baseline) for Task 2.

**TABLE 1:** Summary of indicators computed from raw physiological signals. Identical indicators computed from both ECG and respiration (RESP) signals are grouped together. IBIs refers to interbeat intervals (ECG) and BBs refers to breath‐to‐breath cycles (RESP)

Signal	Indicator	Domain	Description
EDA	Mean raw EDA level		The mean value of filtered EDA signal
	Min raw EDA value		The minimum value of filtered EDA signal
	Max raw EDA value		The maximum value of filtered EDA signal
	Std raw EDA value		The standard deviation of filtered EDA signal
	Mean tonic EDA level		The mean value of tonic EDA signal
	Max tonic EDA value		The minimum value of tonic EDA signal
	Min tonic EDA value		The maximum value of tonic EDA signal
	Std tonic EDA value		The standard deviation of tonic EDA signal
	Mean amplitude of NS‐SCRs		The mean amplitude of NS‐SCRs (computed from phasic EDA signal)
	Frequency of NS‐SCRs		The number of NS‐SCRs per minute (computed from phasic EDA signal)
ECG/RESP	Mean rate	Time‐domain	The mean number of cardiac cycles per minute
	Mean		The mean time of IBIs/BBs
	Median		The median of the absolute values of the successive differences between adjacent IBIs/BBs
	MAD		The mean absolute deviation of IBIs/BBs
	SD		The standard deviation of IBIs/BBs
	SDSD		The standard deviation of the successive differences between adjacent IBIs/BBs
	CV		The coefficient of variation, i.e., the ratio of SD divided by Mean
	mCV		Median‐based coefficient of variation, i.e., the ratio of MAD divided by Median
	RMSSD		The square root of the mean of the sum of successive differences between adjacent IBIs/BBs
	CVSD		The coefficient of variation of successive differences; the RMSSD divided by Mean IBI
	LF	Frequency domain	The spectral power density pertaining to low frequency band (0.04–0.15 Hz)
	HF		The spectral power density pertaining to high frequency band (0.15–0.4 Hz)
	LF/HN		The ratio of LF to HF
	SD1	Non‐linear domain	The measure of the IBIs/BBs spread on the Poincare´ plot perpendicular to the line of identity (short‐term fluctuations)
	SD2		The measure of the IBIs/BBs spread on the Poincare´ plot along the line of identity (long‐term fluctuations)
	SD2/SD1		The ratio between long and short term fluctuations of IBIs (SD2 divided by SD1)
	ApEn		Approximate entropy
ECG	pNN50	Time‐domain	The proportion of successive IBIs greater than 50 ms, out of the total number of IBIs
	pNN20		The proportion of successive IBIs greater than 20 ms, out of the total number of IBIs
	TINN		The baseline width of IBIs distribution obtained by triangular interpolation
	HTI		The HRV triangular index, measuring the total number of IBIs divided by the height of the IBIs histogram
	IQR		The interquartile range (IQR) of the RR intervals
	SDNNI1(2)		The mean of the standard deviations of RR intervals extracted from 1(2)‐minute(s) segments of time series data
	SDANN1(2)		The standard deviation of average RR intervals extracted from 1(2)‐minute(s) segments of time series data

	VHF	Frequency domain	Variability, or signal power, in very high frequency (0.4–0.5 Hz)
	LFn		The normalized low frequency, obtained by dividing the low frequency power by the total power
	HFn		The normalized high frequency, obtained by dividing the low frequency power by the total power
	LnHF		The log transformed HF
	CSI	Non‐linear domain	The Cardiac Sympathetic Index
	CVI		The Cardiac Vagal Index
	CSI modified		The modified CSI was obtained by dividing the square of the longitudinal variability by its transverse variability.
	S		Area of ellipse described by SD1 and SD2
	SampEn		Sample entropy
	PIP		Percentage of inflection points of the RR intervals series.
	IALS		Inverse of the average length of the acceleration/deceleration segments
	PSS		Percentage of short segments
	PAS		Percentage of IBIs in alternation segments
	GI		Guzik's Index
	SI		Slope Index
	AI		Area Index
	PI		Porta's Index
	C1d/C1a		Indices of respectively short‐term HRV deceleration/acceleration
	SD1d/SD1a		Short‐term variance of contributions of decelerations and accelerations
	C2d/C2a		Indices of respectively long‐term HRV deceleration/acceleration
	SD2d/SD2a		Long‐term variance of contributions of decelerations and accelerations
	Cd/Ca		Total contributions of heart rate decelerations and accelerations to HRV
	SDNNd/SDNNa		Total variance of contributions of heart rate decelerations and accelerations to HRV
	DFA alpha1 (2)		The monofractal detrended fluctuation analysis of the HR signal corresponding to short(long)‐term correlations
	DFA alpha1 (2) ExpRange		Range of singularity exponents, corresponding to the width of the singularity spectrum from the monofractal detrended fluctuation analysis of the HR signal, corresponding to short(long)‐term correlations
	DFA alpha1 (2) DimRange		Range of singularity dimensions, corresponding to the height of the singularity spectrum from the monofractal detrended fluctuation analysis of the HR signal, corresponding to short(long)‐term correlations
	DFA alpha1 (2) ExpMean		Mean of singularity exponents from the monofractal detrended fluctuation analysis of the HR signal, corresponding to short(long)‐term correlations
	DFA alpha1 (2) DimMean		Mean of singularity dimension from the monofractal detrended fluctuation analysis of the HR signal, corresponding to short(long)‐term correlations
	ShanEn		Shannon entropy
	FuzzyEn		Fuzzy entropy
	MSE		Multiscale entropy
	CMSE		Composite multiscale entropy
	RCMSE		Refined composite multiscale entropy
CD	Correlation dimension	

	HFD		Higuchi's Fractal Dimension of the HR signal
	KFD		The Katz's Fractal Dimension of the HR signal
LZC	The Lempel‐Ziv complexity of the HR signal	
RESP	Mean amplitude	Time domain	The mean respiratory amplitude.
	Phase Duration Inspiration		The average inspiratory duration
	Phase Duration Expiration		The average expiratory duration
	Phase Duration Ratio		The inspiratory‐to‐expiratory time ratio (I/E)
RSA	Mean (P2T)		Mean of RSA estimates (peak‐to‐trough method)
	Mean Log (P2T)		The logarithm of the mean of RSA estimates (peak‐to‐trough method)
	SD (P2T)		The standard deviation of all RSA estimates (peak‐to‐trough method)
	Mean (Gates)		Mean of RSA estimates (Gates method)
	Mean Log (Gates)		The logarithm of the mean of RSA estimates (Gates method)
	SD (Gates)		The standard deviation of all RSA estimates (Gates method)
	PorgesBohrer		The Porges‐Bohrer estimate of RSA, optimal when the signal to noise ratio is low, in ln(msˆ2)

The scikit learn machine‐learning framework (Pedregosa et al., 2011) was used to implement the classification pipeline in Python (feature normalization, selection, training, and evaluation of classifiers). For classifiers requiring features with equal ranges, features were normalized between the first quartile and the third quartile of data distribution of each feature (RobustScaler method from scikit learn; Pedregosa et al., 2011). To reduce the dimensionality and select only relevant features for the classification, the best features were selected during a univariate feature selection process (SelectKBest method from scikit learn; Pedregosa et al., 2011). The 20 highest scoring features according to the f‐value calculated by analysis of variance (ANOVA) were considered for training the model. Three machine learning algorithms were trained to predict drivers’ condition: random forest (RF), k‐nearest neighbors (KNN) and a neural network with one hidden layer (NN). This choice was based on results from previous studies in the field (Darzi et al., 2018; Solovey et al., 2014; Son et al., 2013).

To train them and validate the results, a 5‐repeated 4‐fold cross‐validation approach was applied to prevent classifiers from overfitting the data and obtain results that reflect the real performance of the model (Hastie et al., 2009). At each iteration, the dataset was split into a training set (80%) and a test set (20%). The training set was split into *k* = 4 folds due to the reduced size of the dataset (only 60 training examples). Classifiers were trained using data from three subsets and then validated on the remaining subset. This process was repeated four times, with each subset acting as the validation set once. A grid search technique was employed in parallel to search for the best set of hyperparameters. It consists of predefining a range of values to test for each hyperparameter. At each iteration of the training procedure, the classifier performed the cross‐validation procedure with all possible combinations of parameters. The model obtaining the best score during the training (grid search) for a given set of hyperparameters was used for evaluation on the test set. Reported results are the mean f1‐score achieved by each classifier on the test set over five iterations.

A post hoc analysis determined the most important features in the classification process, using the SHAP (SHapley Additive exPlanations) library in Python (Lundberg & Lee, 2017). For each feature, it assigns an importance value for a particular prediction, called SHAP value. A list of the most significant features in descending order (ordered by absolute mean of SHAP value) was extracted, for each condition predicted in this study (presence of passenger and relaxation). The SHAP values were calculated on models trained with the three physiological signals.

### 2.7 Statistical analysis and exclusion of participants

The subjective affective state and the physiological data were analyzed using two factorial repeated measures ANOVA for investigating the effect of time, performance of relaxation, and presence of a passenger. To do that, the mean tonic EDA level, the heart rate, and the breathing rate, as well as the positive and negative affect, during the different periods of the experiment (baseline, relaxation, and driving) were used. If Mauchly's test of sphericity indicated that the assumption of sphericity was violated (*p* < 0.o5), Greenhouse‐Geiser sphericity corrections were applied. Post hoc tests with Bonferroni correction were done when the effect of time was significant. The physiological signals of five participants could not be processed, due to bad quality signal (3) and data parsing issues (2). Hence, the statistical analysis was run with the data corresponding to 55 participants.

## 3 RESULTS

### 3.1 Affective state of drivers

The repeated measures ANOVA indicated a significant effect of time on positive affect (*F* (2, 108) = 28.11, *p *< 0.001, *η* ^2^ = 0.10). Post hoc tests indicate that compared to baseline, positive affect decreased after the relaxation phase (*t* (55) = 5.80, *p* < 0.001) and increased after driving (*t* (55) = −7.02, *p* < 0.001) regardless the condition of participants. The presence of a passenger, performance of relaxation, and all interaction effects were not significant (*p* > 0.05).

Besides, no significant effect of time was found on negative affect (*F* (1.336, 71.06) = 3.30, *p *> 0.05, *η* ^2^ = 0.02). The presence of a passenger, performance of relaxation, and all interaction effects were not significant either (*p* > 0.05).

### 3.2 Physiological state of drivers

#### 3.2.1 Mean EDA tonic level

Data analysis showed a significant main effect of time on the mean EDA tonic level (*F* (1.63, 83.33) = 39.52, *p *< 0.001, *η* ^2^ = 0.05). Post hoc tests revealed that drivers’ EDA was higher while driving compared to relaxation (*t* (51) = −8.14, *p* < 0.001) and baseline (*t* (51) = −7.16, *p* < 0.001) periods.

**FIGURE 2:**
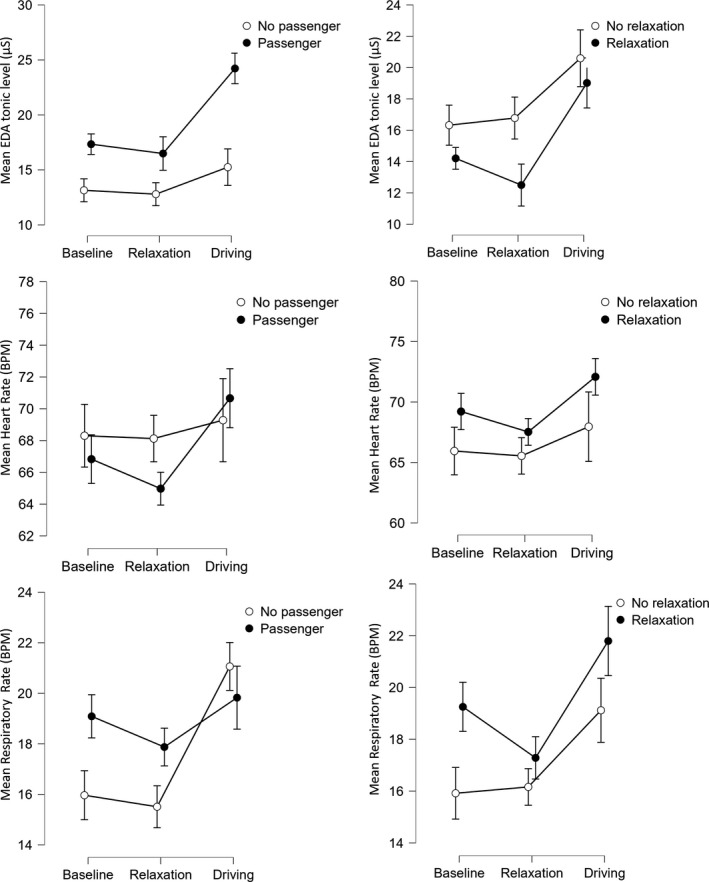
Evolution of participants’ physiological indicators over time. Left: Influence of passenger; Right: Influence of relaxation

Data analysis also showed a significant main effect of presence of passenger on the mean tonic EDA level (*F* (1, 51) = 5.20, *p *< 0.001, *η* ^2^ = 0.08). Figure 2 shows that participants who drove with a passenger had a higher mean EDA tonic level.

Besides, the analysis also showed a significant interaction effect of time and presence of passenger on EDA (*F* (1.63, 83.33) = 10.61, *p *< .001, *η* ^2^ = .01). Post hoc tests revealed that the mean tonic EDA level was significantly higher for the experimental group while driving than for the control group (*t* (51) = −3.47, *p* < .05), while it was not different between groups during the relaxation (*t* (51) = −1.46, *p* > 0.05) and the baseline (*t* (51) = −1.63, *p* > 0.05) phases.

Otherwise, relaxation and other interaction effects were not significant on EDA (*p* > 0.05).

#### 3.2.2 Mean heart rate

Data analysis showed a significant main effect of time on the mean heart rate of individuals (*F* (1.43, 72.85) = 8.04, *p <* 0.01, *η* ^2^ = 0.01), after sphericity corrections. Post hoc tests revealed that drivers’ heart rate was higher while driving compared to relaxation (*t* (51) = −3.90, *p* < 0.001) and baseline (*t* (51) = −2.75, *p* < 0.05) periods.

The presence of a passenger, performance of relaxation, and all interaction effects were not significant on mean heart rate (*p* > 0.05).

#### 3.2.3 Mean respiratory rate

Data analysis showed a significant main effect of time on the mean respiratory rate (*F* (1.61, 82.02) = 37.52, *p *< 0.001, *η* ^2^ = 0.04). Post hoc tests revealed that drivers’ respiratory rate was higher while driving compared to relaxation (*t* (51) = −8.26, *p* < 0.001) and baseline (*t* (51) = −6.38, *p* < 0.001) periods.

Besides, the analysis also showed a significant interaction effect of time and presence of passenger on respiratory rate (*F* (1.61, 82.02) = 13.03, *p *< 0.001, *η* ^2^ = 0.01). Post hoc tests revealed that compared to baseline, the respiratory rate was higher for the control group while driving (*t* (51) = −7.80, *p *< 0.001), but it was not significantly different for the experimental group (*t* (51) = −1.16, *p* > 0.05).

The interaction effect of time and relaxation was marginally significant on drivers’ respiratory rate (*F* (1.61, 82.02) = 3.14, *p *= 0.06, *η* ^2^ = 0.004). Post hoc tests revealed that between the baseline and relaxation phases, the respiratory rate did not change for the control group (*t* (51) = −0.38, *p* > 0.05), while it significantly decreased for the experimental group (*t* (51) = 3.02, *p* < 0.05).

The effect of passenger and relaxation alone, as well as other interaction effects were not significant mean respiratory rate (*p* > 0.05).

### 3.3 Classification of drivers’ condition

#### 3.3.1 Task 1: Presence of passenger while driving

For all possible combinations of physiological signals, Table 2 shows the best performance achieved by the model to predict drivers’ condition while driving (passenger vs. no passenger). The best performance was achieved with the EDA and respiration signals as inputs of a KNN classifier (90% accuracy, *SD* = 9%). The second‐best result was achieved with the three signals as input signals of a RF classifier (86% accuracy, *SD* = 13%).

**TABLE 2:** Best performance achieved for each combination of selected signals to predict the presence of a passenger. Bold values indicate the best score (with classifier) across all combinations of signals

Selected signal(s)	Best classifier	Best score
EDA	RF	0.73 (0.03)
ECG	KNN	0.75 (0.10)
RESP	RF	0.80 (0.07)
EDA + ECG	KNN	0.83 (0.12)
**EDA + RESP**	**KNN**	**0.90 (0.09)**
ECG + RESP	RF	0.82 (0.09)
EDA + ECG + RESP	RF	0.86 (0.13)

#### 3.3.2 Task 2: Practice of relaxation

Table 3 presents the best performance achieved by the model to predict the performance of relaxation (relaxation vs. audiobook), based on features calculated during the relaxation phase. The best performance was achieved with the three signals as input of a NN classifier (80% accuracy, *SD* = 9%). The second‐best result was achieved by a KNN classifier using EDA and ECG signals (78% accuracy, *SD* = 13%).

**TABLE 3:** Best performance achieved for each combination of selected signals to predict pre‐ driving relaxation, based on features calculated during the relaxation phase. Bold values indicate the best score (with classifier) across all combination of signals

Selected signal(s)	Best classifier	Best score
EDA	RF	0.63 (0.12)
ECG	KNN	0.70 (0.13)
RESP	NN	0.78 (0.14)
EDA + ECG	KNN	0.78 (0.13)
EDA + RESP	NN	0.75 (0.10)
ECG + RESP	RF	0.74 (0.14)
**EDA + ECG + RESP**	**NN**	**0.80 (0.09)**

#### 3.3.3 Task 3: Practice of relaxation based on features during the driving phase

Table 4 shows the performance of the model to predict drivers’ condition during the relaxation phase (relaxation vs. audiobook), based on physiological features computed from the driving session. The best score achieved for each combination of signals is summarized in Table 4. The best accuracy was achieved by the KNN classifier using EDA and ECG as inputs (70% accuracy, *SD* = 16%).

**TABLE 4:** Best performance achieved for each combination of selected signals to predict pre‐ driving relaxation, based on features calculated during the driving phase. *Best accuracy* column is the mean (with standard deviation) and *Features* is the number of features used for the classification task. Bold values indicate the best score (with classifier) across all combination of signals

Selected signal(s)	Best classifier	Best accuracy
EDA	RF	0.42 (0.09)
ECG	RF	0.56 (0.13)
RESP	KNN	0.50 (0.10)
**EDA + ECG**	**KNN**	**0.70 (0.16)**
EDA + RESP	NN	0.60 (0.09)
ECG + RESP	RF	0.56 (0.07)
EDA + ECG + RESP	NN	0.64 (0.15)

#### 3.3.4 Most important features for each classification task

**FIGURE 3:**
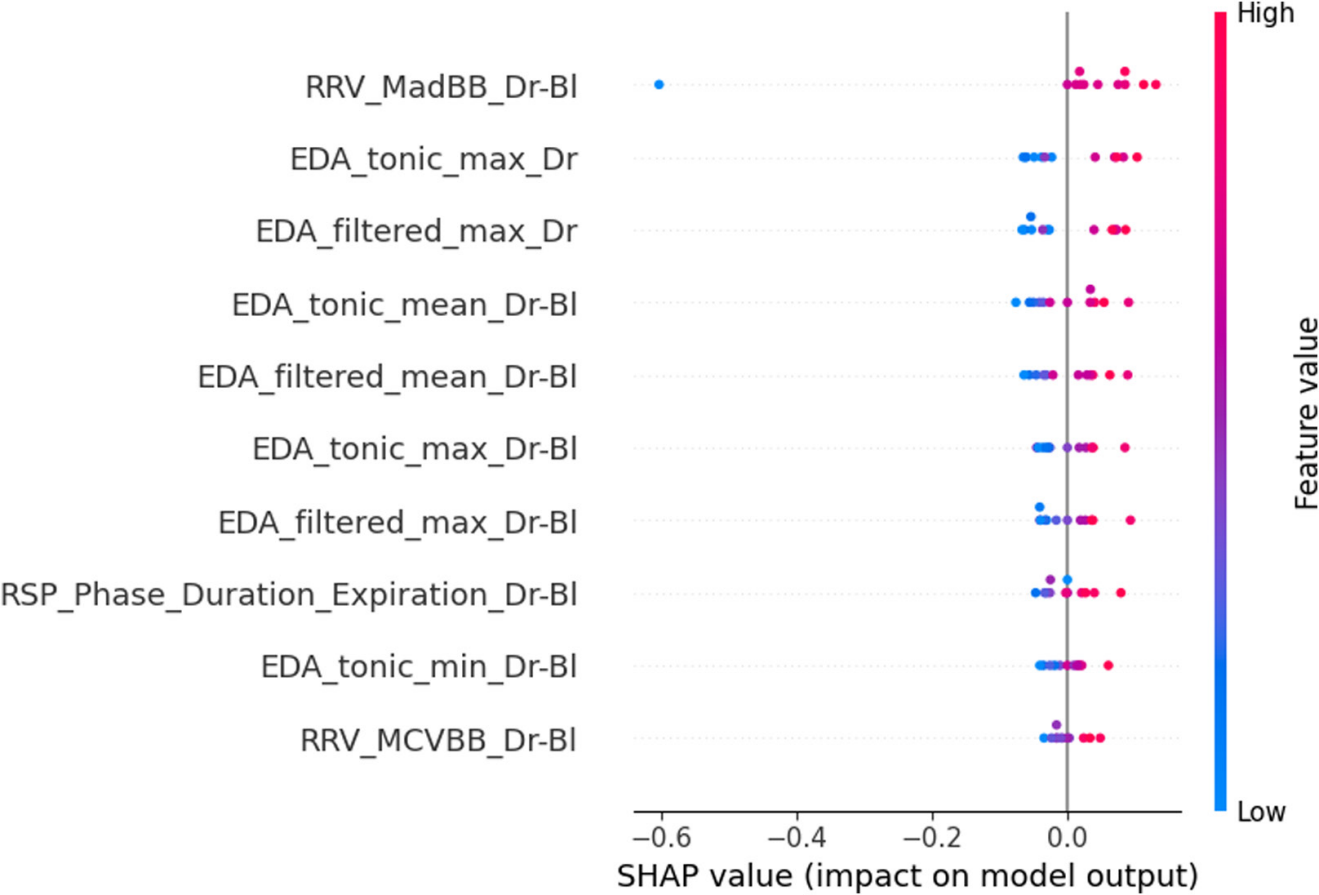
Most important features in the classification process of task 1 (presence of passenger), based on SHAP values calculated on the test set. The meaning/description of each feature can be found in Table 1. Bl, with baseline correction; EDA, electrodermal activity; RRV, respiratory rate variability

**FIGURE 4:**
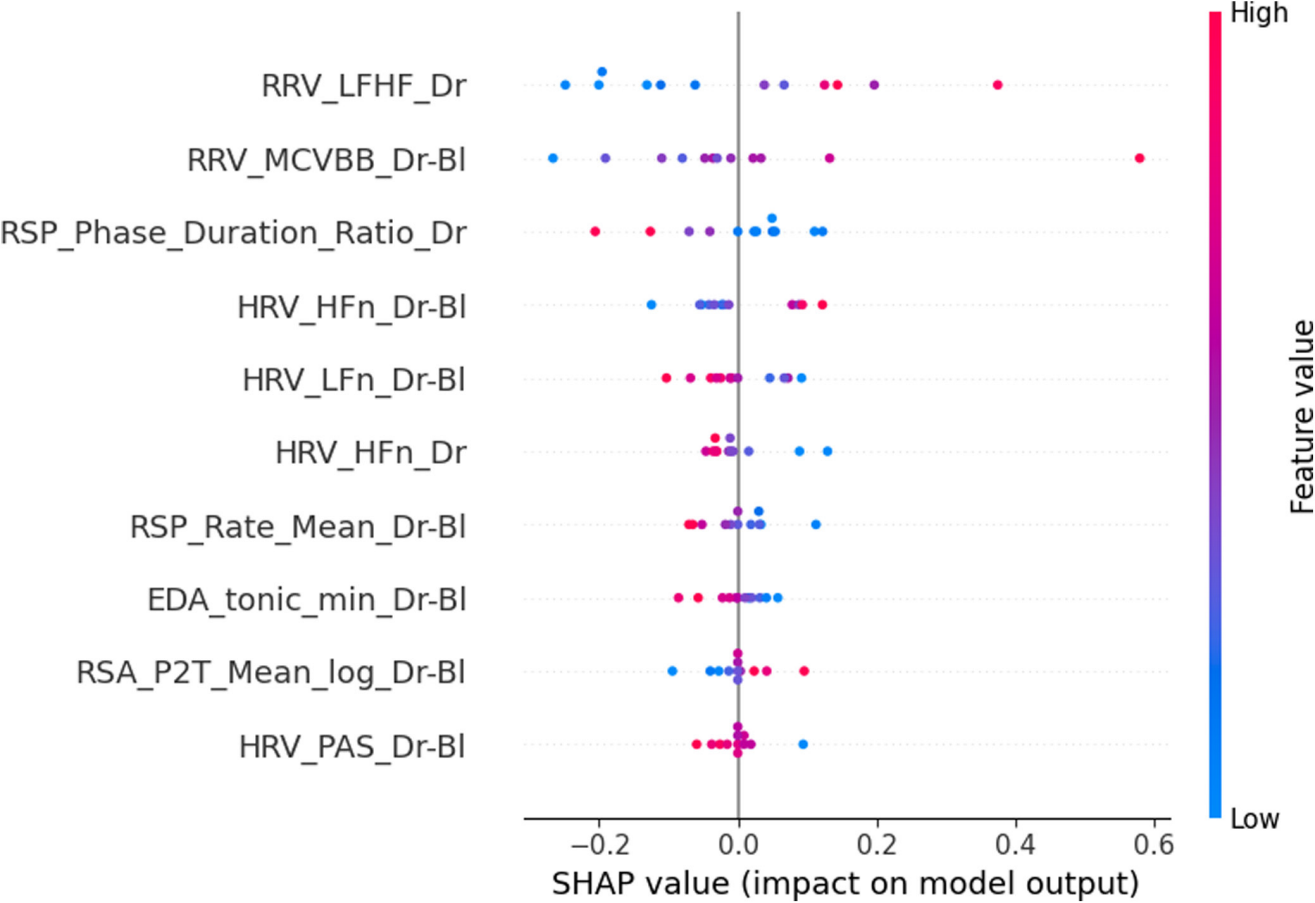
Most important features in the classification process of task 2 (relaxation practice), based on SHAP values calculated on the test set. The meaning/description of each feature can be found in Table 1. Bl, with baseline correction; HRV, heart rate variability; RRV, respiratory rate variability; RSP, respiration

**FIGURE 5:**
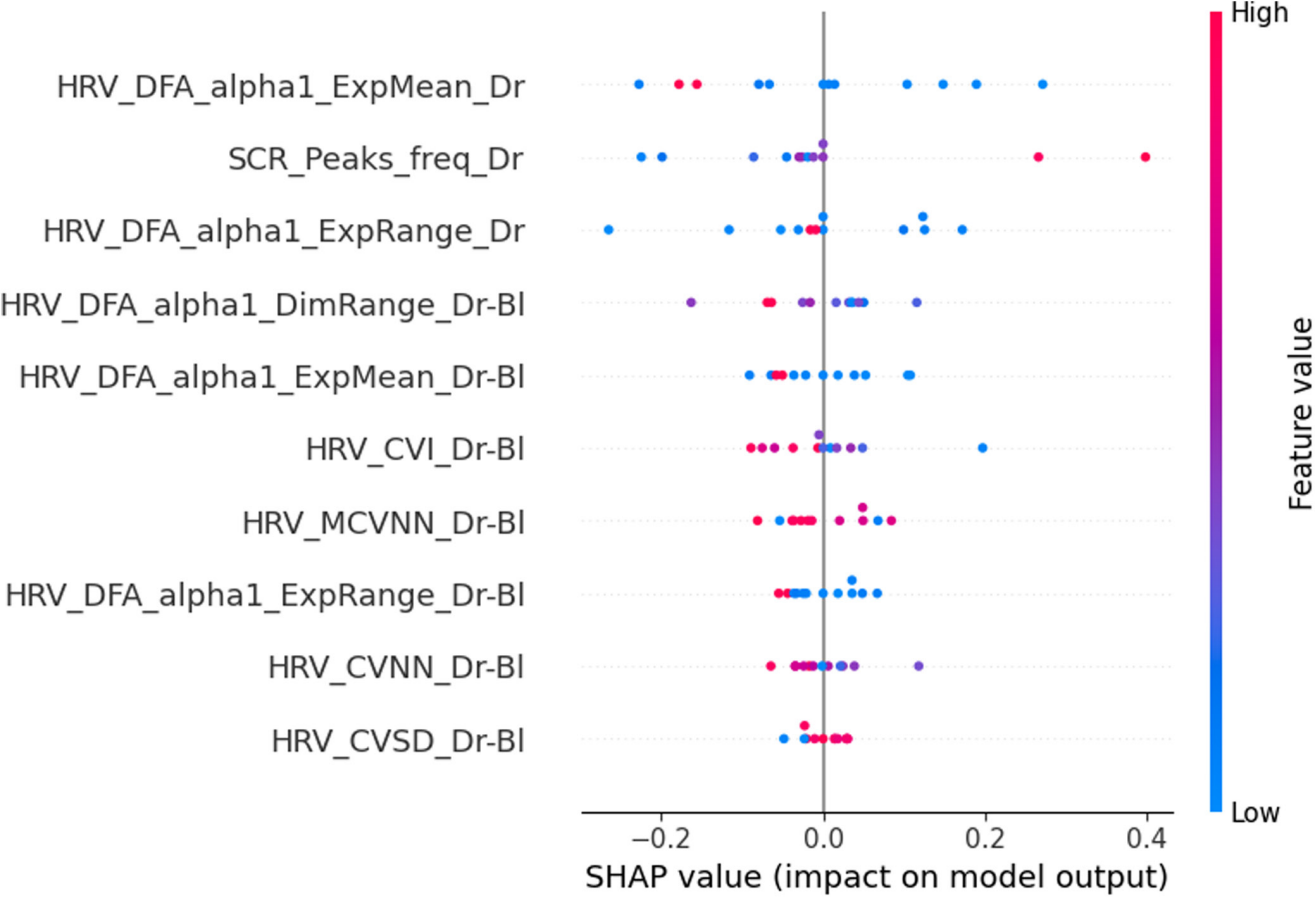
Most important features in the classification process of Task 3 (relaxation practice based on features calculated during the drive), based on SHAP values calculated on the test set. The meaning/description of each feature can be found in Table 1. Bl, with baseline correction; HRV, heart rate variability; SCR, skin conductance response

To understand the decision of the models and to find out the most relevant features as indicators of the physiological activation induced by the presence of a passenger and relaxed state, Figures 3, 4, 5 show the 10 most impacting in the respective classification Tasks 1, 2, and 3. Each point on the graph is the Shapley value of a feature for a given participant. Points to the right of the median axis most influence the model's decision to predict the experimental group condition (passenger and relaxation), while those to the left influence most the decision for predicting the control condition (no passenger and audiobook).

## 4 DISCUSSION

### 4.1 Change of physiological and affective state over time

Data analysis revealed that compared to baseline, the drivers’ positive affect decreased after relaxation and then increased after driving. Participants may have felt that the relaxation/audiobook phase was boring while driving in the simulator was more entertaining. However, no change in negative affect was observed during the experiment. Data analysis also showed that the physiological state of participants changed during the experiment. Participants’ mean tonic EDA level, heart rate, and respiratory rate increased significantly during the driving phase compared to baseline and relaxation, demonstrating the stimulating nature of this task (Healey & Picard, 2005).

### 4.2 Presence of passenger

#### 4.2.1 Change in physiological and affective state.

Data analysis showed that the presence of a passenger was linked with an increase in the mean tonic EDA level of participants, but not on the mean heart rate or respiratory rate. Since EDA can be considered related to arousal (Boucsein, 2012), the results show that the presence of a passenger is associated with an increase in arousal. Interestingly, the respiratory rate increased for the control group while driving, compared to baseline, but it was not the case for participants driving with a passenger. The drivers’ vocal interaction with the passengers may have affected their breathing pattern and hence reduced the increase in respiratory rate of the experimental group. Besides, the presence of a passenger did not have any effect on drivers’ affective state. It indicates that drivers did not feel subjectively affected by the presence of a passenger, whereas it affected their physiological activation.

#### 4.2.2 Classification and the most relevant features

Results indicate that the presence of a passenger can be detected with 90% accuracy, based on 20 features extracted from EDA and respiration signals. The model has consistently achieved 70% accuracy in predicting the presence of a passenger, regardless of the physiological signals selected. Sensor fusion improved the accuracy of the model. According to the results obtained in this study, the accuracy achieved is lower than that achieved in the works of Healey and Picard (2005) and Chen and colleagues (2017). The latter showed that signals of ECG and EDA collected from the foot yielded higher performances than the EDA signal collected from the hand. Different features and classification procedures were used in our study, so it is difficult to compare the results and argue that the physiological activation induced by the presence of a passenger is more difficult to classify than that induced by the driving environment.

Post hoc analysis of feature importance in the classification process suggests that features calculated from the EDA and respiration signals should be used to predict the presence of a passenger with machine learning techniques. The presence of a passenger is associated higher skin conductivity and longer exhalations, likely due to the vocal interaction between the driver and the passenger (see Figures 2 and 3). Results are consistent with the findings of Healey and Picard (2005), who found that the mean EDA level correlated most with stress in the context of driving.

In summary, the model implemented in this work was able to detect the presence of a passenger in a controlled environment, mainly due to an increase in drivers’ arousal (measured through EDA indicators). In real driving situations, a significant increase of arousal could be detected in young drivers, using machine learning models as the one proposed in this work. Young drivers could be warned when an increase in arousal is detected, which could help prevent them from taking more risks behind the wheel (Centifanti et al., 2016; Chen, 2000). The physiological changes detected by the model can be interpreted as a form of social stress, due to being observed while performing a task by an unknown individual (Schrier, 1992).

### 4.3 Practice of relaxation

#### 4.3.1 Change in physiological and affective state

Interestingly, the results of relaxation before driving did not show the expected effect pattern. No effect of relaxation was found on drivers’ physiological and affective state. Only the manipulation check revealed that participants following a mindfulness exercise reduced their respiratory rate compared to participants listening to an audiobook, although this effect was only marginally significant. However, this potential beneficial effect did not carry over the driving situation, regarding the statistical analysis. This remains to be confirmed by the classification task.

#### 4.3.2 Classification results and relevant features of a relaxed state

In the classification Task 2, relaxation could be predicted with 80% accuracy using all signals as input to a neural network classifier. Again, sensor fusion allowed the model to perform better. Considering each physiological signal alone, EDA showed the lowest accuracy. Classification task 3 was the most challenging because no physiological data collected during the relaxation phase was used. Still, the model achieved 70% accuracy using EDA and ECG as input signals. This shows that the effect of relaxation carried over the driving phase, in contrast with the statistical analysis above. This shows that it is relevant to use machine learning techniques in some contexts, even when the statistical analysis is not significant. However, the results show that the model still has difficulty predicting the condition of subjects who performed relaxation before driving. The physiological change on the drivers’ state while driving was probably not significant enough. This implies that a longer phase of mindfulness meditation might be necessary before driving if one wants to benefit from the effect of such a stress management technique while driving.

The post hoc analysis of the significance of features revealed that the participants in the manipulation group correctly practiced the relaxation. Indeed, they exhale longer, which led to a decrease in their breathing rate (see Figures 2 and 4). The analysis showed that features such as the ratio of low to high frequencies of the respiratory signal, frequency measures in the low‐ and high‐frequency bands of the HRV (with baseline correction) and the minimum level of skin conductance (see Figure 4) were among the most useful features for predicting a relaxed state. Besides, it seems that the effect of pre‐driving relaxation might mainly be observed via heart rate variability indicators in the nonlinear domain, calculated by (multifractal) detrended fluctuation analysis (DFA, see Table 1 and Figure 5).

### 4.4 Limitations

#### 4.4.1 Presence of passenger and young drivers

In this study, the model can detect the presence of a passenger in a simulated environment. However, it has yet to be confirmed that the model would also be able to do so in a more complex real‐world driving environment, including other stressors (such as bad weather or heavy traffic). It is very likely that this model detects some mere physiological activation but not specifically the presence of a passenger. Future research might address the question whether different stressors could be distinguished based on specific physiological reactions. As mentioned earlier, results showed that respiration features were useful in predicting the presence of a passenger. However, vocal interaction between confederates and participants might have played a role in the difference in breathing behavior between the two experimental groups. For further research, the mere presence of a passenger and the vocal interaction between the driver and the passenger should be carefully controlled in separate experimental condition.

Another limitation of this study may be the focus on young adults. While this work is an interesting jigsaw piece for understanding the physiological responses of young drivers related to the presence of a passenger, it would be interesting to learn if a similar effect could be reported for older, more experienced drivers. Given that they appear to show an opposite effect pattern to young drivers (i.e., fewer accidents with a passenger present (Williams et al., 2007)) it remains unclear which role age and driving experience play in moderating the relationship between arousal and risky driving behavior. Future experimental studies should address this issue by comparing the consequences of passenger presence among drivers of different age groups and driving experiences.

#### 4.4.2 Experience with meditation

Concerning the interesting but unexpected findings on relaxation (e.g., higher increase in physiological indicators while driving), the data indicate that there was an effect of the intervention. The term relaxation was chosen in this research, even though the intervention applied in this study was listening to a guided mindfulness meditation exercise. It can be argued that (mindfulness) meditation is considered a very specific technique that requires extensive training and experience (e.g., Barinaga (2003); Moore and Malinowski (2009)). However, in the present study, most participants had no prior experience with meditation techniques. Although a guided‐mindfulness meditation training was used in this study, especially those new to meditation, cannot be expected to enter a self‐regulatory meditative state that includes full control of the components of enhanced attention control, improved emotion regulation, and altered self‐ awareness (c.f. Tang et al. (2015)).

In addition, some participants may have been more interested in practicing relaxation than others. Thus, the effect of relaxation may have been reduced on the physiological state of those who lost interest in the relaxation task. Future studies that attempt to replicate the findings of this experiment should consider the experience of participants in meditation to assess its potential moderating influence on the long‐term consequences of meditation before driving.

#### 4.4.3 Experimental setting and procedure

For the experimental settings of that study, driving in a simulator could have reduced the sense of danger compared to a real‐world driving situation and thus the behavior exhibited in this artificial environment could, despite the highly immersive nature of the installation, be different from behavior in a real‐world environment as it was the case in the study of Healey and Picard (2005). Although this lack of ecological validity in driving simulator studies might be considered a limitation, a more valid methodological approach to address this research question in a more natural environment would be linked to severe safety issues and ethical implications. Indeed, it is ethically unjustifiable to conduct experimental studies on research questions in which participants are placed in risky situations. Therefore, a combination of correlational studies referring to real‐world accident statistics data and experimental studies to investigate cause and effect relationships may be the best solution in this area of research.

According to the experimental procedure employed in this study, the practice session was done just before the main driving session. Although it was short, this may have affected the physiological and affective state of the drivers and thus reduced the effect of the relaxation done just before. In other similar studies, the relaxation should be performed just before the main driving period, and the practice in the simulator should be performed at the very beginning of the experimental procedure.

Finally, the same audiobook was used for all participants. Some of them might have been more interested than others by the story. Hence, these participants were possibly more focused on listening the audiobook, which could have affected their physiological state (i.e., increased mental load).

## 5 CONCLUSION

Findings of this piece of research show that drivers experience a higher increase in physiological activation when driving with a passenger, which can be predicted with 90%‐accuracy by a k‐nearest neighbors classifier. A short relaxation phase (10 min) before driving could be recognized with 80%‐accuracy based on three physiological signals. According to the statistical analysis, the potential beneficial effect of relaxation did not subsequently affect the driver's state during driving, although the classification task suggested the opposite. Indeed, a k‐nearest neighbors classifier was able to recognize with 70% accuracy the participants who exercised relaxation before driving, based on the features of heart rate variability and electrodermal activity. In addition, some of the most relevant physiological indicators associated with the presence of a passenger and a relaxed state are proposed in this study. The finding of this study suggest that skin conductivity characteristics should be used to detect physiological activation associated with the presence of a passenger, while cardiac and respiratory variability indicators are better at predicting relaxation. Finally, the results suggest that the effect of relaxation on the driver's state later during driving might be observed via the cardiac variability indicators. In the perspective of making future cars smarter and safer, machine learning models implemented in this study could be used to assess the driver's state continuously, by selecting the physiological features suggested in this study.

## CONFLICT OF INTEREST

The authors declare that the research was conducted in the absence of any commercial or financial relationships that could be construed as a potential conflict of interest.

## AUTHOR CONTRIBUTIONS

AS, OAK, EM, LA, and MW found the research gap and initiated the study. QM and AS made the experimental design and managed data collection. QM and MC designed the driving scenario. QM and EDS implemented the pipeline to calculate physiological indicators from raw signals and do the classification tasks. All authors participated with the writing and revising processes.
